# Lipase catalyzed synthesis of antimicrobial andrographolide derivatives

**DOI:** 10.1016/j.dib.2018.03.103

**Published:** 2018-03-28

**Authors:** Harshal S. Patil, Dipesh D. Jadhav, Ajay Paul, Fayaj A. Mulani, Shrikant J. Karegaonkar, Hirekodathakallu V. Thulasiram

**Affiliations:** a Chemical Biology Unit, Division of Organic Chemistry, CSIR-National Chemical Laboratory, Pune 411008, India; b Academy of Scientific and Innovative Research, Anusandhan Bhawan, 2 Rafi Marg, New Delhi 110001, India; c CSIR-Institute of Genomics and Integrative Biology, Mall Road, New Delhi 110007, India

**Keywords:** Andrographolide, Lipase, Antimicrobial

## Abstract

In this data article we describe screening of various lipases for the regioselective acylation of Andrographolide. Each lipase was screened with seven acyl donors. Amano lipase AK from *Pseudomonas fluorescens* was used for the synthesis of two new acylated andrographolide derivatives. Two new compounds, andrographolide-14-propionate and andrographolide-14-caproate were characterized by various spectral studies. These two derivatives showed more antimicrobial activity than andrographolide.

**Specifications table**

**Table:** 

Subject area	Chemistry
More specific subject area	Lipase-catalyzed acylation
Type of data	Table, experimental procedure, NMR spectra
How data was acquired	NMR (Varian spectrometer-400), High Resolution Mass Spectrometry (Thermo Scientific Q Exactive Quadrupole-Orbitrap Mass Spectrometer)
Data format	Analyzed
Experimental factors	Lipase-catalyzed derivatives were purified and analyzed by HPLC, ESI-HRMS, and NMR.
Experimental features	Regio-selective enzymatic acylation of Andrographilide
Data source location	CSIR-National Chemical Laboratory, Pune
Data accessibility	Data is present in this article.
Related research article	*“in press.”*

**Value of the data**

• The data provide screening results for regio-selective acylation of Andrographolide by using various commercial lipases with various acyl donors.
• In this study we optimized the reaction conditions for Andrographolide acylation by using Amano lipase AK *P. fluorescens lipase* with various acyl donors.
• This study yields structural characterization data of two new compounds: andrographolide-14-propionate and andrographolide-14-caproate.
• The synthesized Andrographolide derivatives can be used as antimicrobial agents. Derivatives were more potent antimicrobial agents when compared to Andrographolide (in the related manuscript).

## 1 Data

Data present in this paper describe the screening of commercially available lipases for the synthesis of 14-acylated andrographolide derivatives (Table 1).

**Table 1:** Screening of various lipases with acyl donors for acylation of Andrographolide.

**Sr. no.**	**Enzyme**	**Source**	**Acetyl donors**						
			**V1**	**V2**	**V3**	**V4**	**V5**	**V6**	**V7**
1.	Novozyme 435	CAL*-*B	r.	n.r.	r.	n.r.	r.	n.r.	n.r
2.	Immobilized on	CAL-A	n.r.	r	n.r.	r.	n.r.	n.r.	n.r
	Immobead 150								
3.	*B. cepacia*	Amano lipase PS	n.r.	n.r.	n.r.	n.r.	n.r.	n.r.	n.r.
4.	Immobilized on diatomite	Amano lipase PS	r	n.r.	n.r.	n.r.	n.r.	n.r.	r
5.	Amano lipase A	*A. niger*	n.r.	n.r.	r	n.r.	n.r.	n.r.	n.r.
6.	Amano lipase AK	*P. fluorescens*	r	r	r	n.r.	r	n.r.	r
7.	CRL	*C. cylindracea*	n.r.	n.r.	n.r.	n.r.	n.r.	n.r.	n.r.
8.	Type II PPL	*P. pancreas*	n.r.	n.r.	n.r.	n.r.	n.r.	n.r.	n.r.
9.	Lipase type VII	*C. rugosa*	n.r.	n.r.	n.r.	n.r.	n.r.	n.r.	n.r.
10.	Lipase	*P. camemberti*	r.	n.r.	n.r.	n.r.	n.r.	n.r.	n.r.
11.	Lipase	*R. niveus*	r.	n.r.	n.r.	n.r.	n.r.	n.r.	n.r.

V1, Vinyl acetate; V2, Vinyl propionate; V3, Vinyl butyrate; V4, Vinyl decanoate; V5., Vinyl laurate; V6., Vinyl benzoate; V7, Vinyl Trifluroacetate. *B. cepacia, Burkholderia cepacia; C. cylindracea, Candida cylindracea; P. pancreas Porcine pancreas; P. camemberti, Penicillium camemberti; R. niveus, Rhizopus niveus.* n.r. = no reaction. r = reaction. Reaction conditions: 0.1 mmol Andrographolide; 1.0 mmol various vinyl donors; 5 mg various enzyme; 5 ml Acetone; 100 rpm; 6 h.

**Fig. 1:**
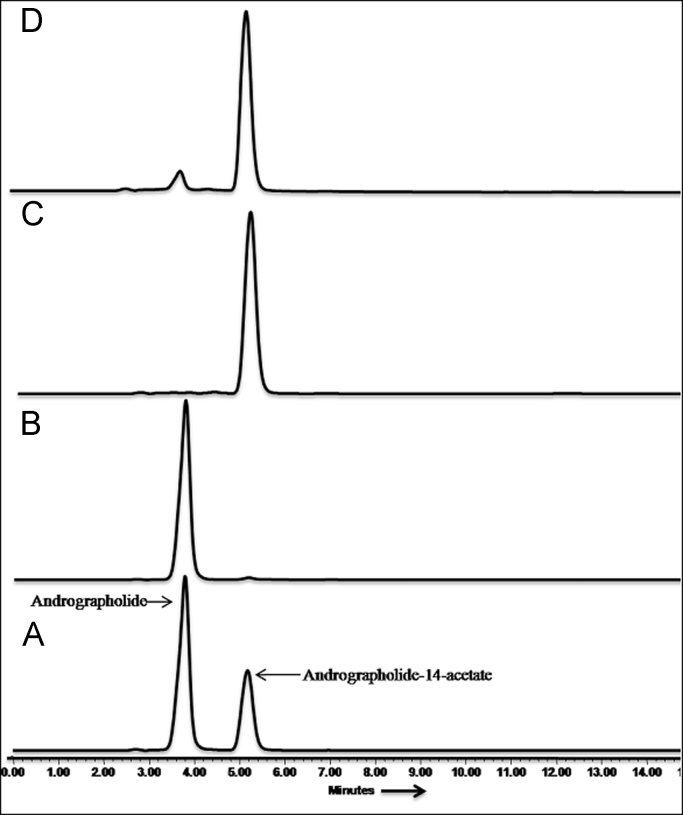
HPLC chromatogram of (A) Reaction mixture Amano lipase AK [0.1 mmol Andrographolide 1.0 mmol vinyl acetate, 5 mg of Amano lipase AK; 55 °C / 1 h; Acetone, 100 rpm, (B) Andrographolide (C) Andrographolide-14-acetate, (D) Co-injection of Andrographolide-14-acetate.

HPLC analysis was performed by using Waters HPLC system with UV–vis detector at 235 nm. Analytical X-Bridge C_18_ column (4.6 × 250 mm, 5 μm) at a flow rate of 1 mL/min. with gradient solvent programme of Acetonitrile (ACN) and water (H_2_O). HPLC grade solvents were purchased from Sigma Aldrich (USA). The substrate conversion and initial reaction rate (*V*_0_) were calculated from the HPLC data (Fig. 1).

Characterization of andrographolide-14-propionate (Fig. S1a-d) and andrographolide-14- caproate (Fig. S2a-d): NMR (^1^H, ^13^C, DEPT) spectra were recorded on Varian spectrometer (400 MHz) and chemical shift values were reported in ppm with respect to the residual solvent signal as the reference. HRMS data were collected on Thermo Scientific Q Exactive Quadrupole-Orbitrap Mass Spectrometer. IR spectra were recorded on Perkin Elmer FT-IR spectrophotometer in CHCl_3_ and optical rotations were determined on JASCO (P-2000), polarimeter using 10 mm cell (*c* in gm/100 ml unit). Acylation position was determined by comparison of the chemical shifts of the acylated product with andrographolide. The structure of products formed was confirmed by comparing with earlier reports [1], [2].

**Fig. 2:**
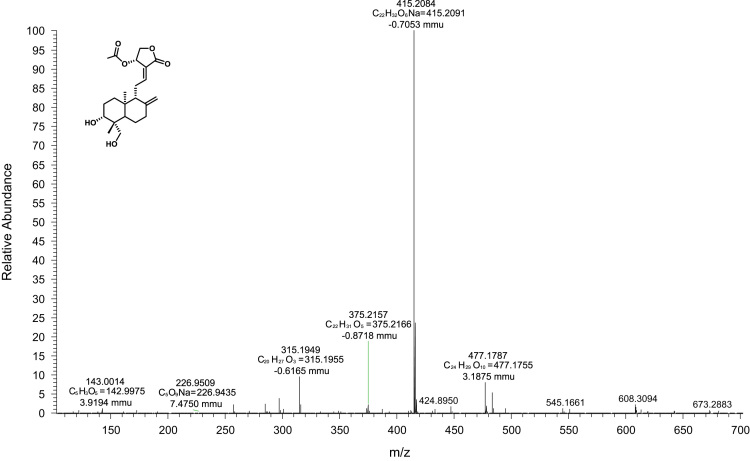
High-resolution mass spectra of andrographolide-14-acetate (2).

Zeiss Axiovert apotome microscope equipped with an AxioCam camera and oil-immersion objective lens (64x) was employed to record the images and were processed with Axiovision 4.7 software. (Fig. 2A in main manuscript) Leica Stereoscan 440 was operated at an accelerating voltage of 15 kV with pressure 5.587E-4 Pa, current 0.14 nA and WD 9.9–10.0 mm det EDT. The pre-scanned samples were sputter coated with 10 nm thick layers of gold nanoparticles by using a Polaron SC 6420 sputter coater. The neat and irradiated samples were sputter coated without any pre-treatment or sample preparation (Fig. 2B in main manuscript).

## 2 Experimental design, materials, and methods

### 2.1 General procedure for lipase-catalyzed acylation of Andrographolide

In general experimental conditions, 0.1 mmol andrographolide, 1.0 mmol vinyl esters, and 5 mg enzyme were dissolved in 3 mL of acetone/ethyl acetate / vinyl acetate / methanol / tetrahydrofuran and incubated in a shaking incubator, as reported earlier [1], [2]. After a specific time of interval, aliquots were drawn from the reaction mixture, filtered through 0.45 µm syringe filter and diluted with methanol for HPLC analysis. The initial water activity (*a*_*w*_) of the reaction (the lipase with the substrates) was controlled in a different saturated salt solution at 25 °C by gaseous equilibrium in separate closed containers as reported earlier [3], [4]. Reaction with the predetermined condition without enzyme served as a negative control, which did not detect the acylated product. All experiments were performed in triplicate, and the average results with standard deviation are reported. For characterization of product formed in a general experiment, 0.1 mmol andrographolide, 1 mmol vinyl esters dissolved in acetone and 90 mg of Amano lipase AK from *Pseudomonas fluorescens* was incubated at 55 °C in a shaking incubator set at 100 rpm for 5 h. The reaction mixture was filtered to get rid of lipase and filtrate was concentrated under vacuum. The obtained residue was further purified by silica gel column chromatography with acetone-petroleum ether mixture as solvent system.

### 2.2 Anti-bacterial activity

The anti-bacterial activity of andrographolide and its derivatives was determined in a 96-well micro titer plate using broth micro-dilution method as reported earlier [2]. The activity was calculated in a total volume of 100 µL containing the test compounds (final concentration range between 0.25–128 μg/mL) and the bacterial suspension (final concentration of 2 × 10^5^ cfu/mL). An assay with Gentamicin sulphate and Benzamide sodium served as positive controls. The experiment was performed in triplicates independently along with growth and sterility controls in each set. Then the micro titer plate was incubated at 37 °C for 20 h. The growth inhibition was visualized and the minimum inhibitory concentration (MIC) was determined as the lowest concentration of compound required for complete growth inhibition.

### 2.3 Antifungal activity

Andrographolide and its derivatives were tested for their antifungal activity against fungal/yeast strains obtained from NCIM. The activity was determined by broth micro dilution method as mentioned above. An assay with Amphotericin B served as a positive control. The assay mixture containing the fungi and test compounds was incubated at 30 °C for 24 h for *A. niger* and 49–72 h for *Candida albicance* and *Rhizopus oryzae*. The growth inhibition was visualized and the MIC was determined as the concentration of compound required for complete growth inhibition.

### 2.4 Hemolysis assay

Hemolytic assay was performed according to protocol reported earlier [5]. Fresh human RBCs (hRBCs) were collected stored with Etylenediaminetetraacetic acid (EDTA). The hRBCs were washed four times with Tris-buffer saline (150 mM NaCl, 10 mM Tris pH 7.2) and diluted to final concentration of 4% v/v. The assay was performed in a sterile 96-well plate at a final volume of 100 μL as follows: an aliquot of 50 μL of hRBCs suspension was added to 50 μL of 2-fold serially diluted (100 μg/mL) andrographolide and its derivatives in the minimum amount of dimethyl sulfoxide (DMSO) diluted with Tris buffer. The plate was incubated at 37 °C for 1.5 h and then centrifuged for 15 mins. at 3000 rpm. The supernatant (50 μL) from each well was transferred to a fresh 96-well plate containing 50 μL of water, and the release of hemoglobin was monitored by measuring the absorbance at 540 nm.

All the experiments were performed in triplicate with hRBC suspensions in Tris buffer served as negative control and in 1% Triton-X comprised as positive control, respectively. The percentage of hemolysis was defined as (A−A_N_)/(A_P_−A_N_) × 100, where A is the absorbance of the test well, A_N_ is the absorbance of the negative control, and A_P_ is the absorbance of the positive control.

### 2.5 Fluorescent staining for assessing cell viability and membrane integrity

A triple staining procedure was followed to assess the viability as well as membrane integrity of the cells as reported earlier [6]. *Escherichia coli* cells were grown in Luria Bertani broth(LB) till an OD_600_ of 0.6 units was attained. The cells were washed twice with 10 mM sodium phosphate buffer (pH 7.4) to remove media and re-suspend in the same buffer. After exposure to inhibitory concentrations of Andrographolide and its derivatives at 37 °C for 60 mins, the bacterial cells were immobilized on poly (L-lysine)-coated glass slides for 50 mins. at 30 °C, followed by addition of 100 μL of DAPI (2 μg/mL in buffer). After 30 mins incubation. at 30 °C, the DAPI solution was washed away and rinsed again with sodium phosphate buffer, followed by addition of 100 μL of FITC (6 μg/ mL in the buffer). It was then incubated at 30 °C for 30 mins. followed by rinsing and addition of 100 μL PI (2 μg/mL in buffer), further continuing incubation at 30 °C for 30 mins. The slide was then washed as described above. The cells without the compounds served as controls. Fluorescent images were recorded using a Zeiss Axio-observer Z1 microscope equipped with an oil-immersion objective (64x) and an AxioCam camera. Image acquisition and processing were performed using Axiovision software.

### 2.6 Bacterial protein leakage assay

To detect leakage of intercellular proteins over time, the bacterial protein leakage assay was performed as reported earlier [7]. The cells were washed with phosphate buffer to remove any extracellular proteins present before incubating with test compounds. The cells were then incubated with test compounds at concentrations double the MICs. at 37 °C for 2 h. At periodic time interval of 30 mins. cell suspension was centrifuged at 7000 g and supernatant collected was used to determine the protein content. The protein concentration in the supernatant was determined by Bradford's assay [8]. All the experiments were performed independently in triplicates. The amount of protein in the supernatant was determined by extrapolating `values in the Bovine Serum Albumin standard curve.

### 2.7 Scanning electron microscopy (SEM)

To observe the morphological changes in bacterial cells, SEM images were recorded for *E. coli* treated with the test compounds. The samples for recording SEM images were prepared as reported earlier [9]. The cells were grown in LB media to an OD_600_ of 0.6 units. and then washed with 10 mM sodium phosphate buffer (pH 7.4) twice, to remove media. The cells were re-suspended in same buffer and treated with MICs of andrographolide and its derivatives at 37 °C for 60 mins., The cells were washed twice with buffer and then pelleted down by centrifugation at 3000 rpm for 20 mins. The cells were fixed with 1% glutaraldehyde in PBS for 30 mins.and re-suspended again. A drop containing the bacteria was deposited onto a silicon wafer and dried overnight. It was then coated with gold and observed in scanning electron microscope.

## 3 Material

### 3.1 Chemical and biological materials

Andrographolide was isolated from *Andrographis paniculata*. Acyl donors including Vinyl acetate, Vinyl propionate, Vinyl butyrate, Vinyl decanoate, Vinyl laurate, Vinyl trifluroacetate, Vinyl benzoate, lipases including Amano lipase PS *Burkholderia cepacia*, Amano lipase A from *A.niger*, CRL from *Candida cylindracea*, Type II PPL from *Porcine pancreas*, Lipase type VII from *C.rugosa*, Lipase from *Penicillium camemberti*, Lipase from *Rhizopus niveus*, lipase immobilized on Immobead 150 *Candida antarctica* lipase-A (CAL-A), Novozyme 435 from *Candida antarctica* lipase-B (CAL-B), Amano lipase AK source *P. fluorescens* were purchased from Sigma-Aldrich (USA). Fluorescein Isothiocyanate (FITC), 4', 6-diamidino-2-phenylindole (DAPI), Propidium iodide (PI) were purchased from Sigma–Aldrich (USA). Reaction progress was monitored by thin-layer chromatography (TLC) performed on silica gel G-coated plates (0.25 mm, Merk) with methanol: dichloromethane (1:19) as the mobile phase. Migration of the compounds on TLC was visualized by spraying it with a solution of 3.2% anisaldehyde, 2.8% H_2_SO_4_, 2% acetic acid in ethanol, followed by heating for 1–2 mins. Andrographolide derivatives were purified over silica gel (230–400 mesh) column chromatography using acetone/ pet ether gradient. Test microorganisms were obtained from National Collection of Industrial Microorganism (NCIM), CSIR-NCL Pune. Antimicrobial activities were assessed using Gram-positive bacteria *Staphylococcus aureus (NCIM2100), M. luteus (NCIM 2170),* Gram-negative bacteria *E. coli (NCIM 2575), P. fluorescens (NCIM 2638),* fungus *Aspergillus fumigatus (NCIM 902), R. oryzae (NCIM 1299)* and yeast *Candida albicans (NCIM 3471).*

## 4 Analytical and NMR data monoester derivatives of andrographolide

*Andrographolide-14-acetate (2)* values matched with reported data [1], [2].

White solid; m.p. 168.4–170.2 °C; IR (CHCl_3_, cm^−1^) υ_max_ = 1673, 1738, 1760, 3366; HRMS (ESI) *m/z*: [M+Na]^+^ Calcd for C_22_H_32_O_6_ Na, 415.2091, found, 415.2084.

^1^H NMR (400 MHz, CDCl_3_, ppm): δ 6.95−6.98 (td, *J* = 6.84, 1.72 Hz, 1H), 5.87−5.89 (d, *J* = 5.81, 1H), 4.83 (s, 1H), 4.50–4.53 (m, 1H), 4.46 (s, 1H), 4.19–4.22 (dd, *J* = 1.52, 10.99 Hz, 1H), 4.12–4.14 (d, *J* = 11, 1H), 3.61–3.63 (m, 1H), 3.40–3.44 (dd, *J* = 2.65, 10.99 Hz, 1H), 3.34–3.37 (m, 1H), 3.25–3.28 (m, 1H), 2.80 (s, 1H), 2.32–2.41 (m, 4H), 2.14−2.13 (m, 1H), 2.08 (s, 3H), 1.97–2.01 (m, 2H), 1.76–1.80 (m, 2H), 1.62–1.71 (m, 2H), 1.20–1.21 (m, 3H),0.63 (s, 3H).

^13^C NMR (100 MHz, CDCl_3_, ppm): δ 170.53, 169.09, 150.53, 146.67, 123.78, 108.70, 80.28, 71.58, 67.72, 64.06, 55.77, 55.11, 42.77, 38.76, 37.65, 36.93, 28.06, 25.25, 23.63, 22.68, 20.67, 15.07.

*Andrographolide-14-propionate (3)*.

White Solid; m.p. 153.2–155.2 °C; IR (CHCl_3_, cm^−1^) υ_max_ = 1673, 1720, 1735, 3490; HRMS (ESI) *m/z*: [M+1]^+^ Calcd for C_23_H_35_O_6_, 407.2424, found, 407.2428.

^1^H NMR (400 MHz, CDCl_3_, ppm): δ 6.95–6.96 (td, *J* = 6.84, 1.72 Hz, 1H), 5.89–5.91 (d, *J* = 5.81, 1H), 4.82 (s, 1H), 4.53–4.56 (dd, *J* = 6.11, 11.25 Hz, 1H), 4.45 (s, 1H), 4.17–4.19 (dd, *J* = 1.71, 11.0 Hz, 1H), 4.11–4.14 (d, *J* = 11.0 Hz, 1H), 3.40–3.43 (m, 1H), 3.25–3.27 (m, 1H), 2.30–2.40 (m, 6H), 1.91–1.96 (m, 1H), 1.77–1.81 (m, 4H), 1.66–1.68 (m, 1H), 1.19–1.25 (m, 6H), 1.07–1.15 (m, 4H), 0.62 (s, 3H).

^13^C NMR (100 MHz, CDCl_3_, ppm): δ 173.92, 169.15, 150.51, 146.65, 123.71, 108.64, 80.00, 71.59, 67.52, 63.93, 55.61, 55.95, 42.49, 38.62, 37.52, 36.80, 27.83, 27.28, 25.14, 23.60, 22.63, 14.98, 8.89.

*Andrographolide-14-butanoate (4)* values matched with reported data [2].

White needles; m.p. 128.9–130.2 °C; IR (CHCl_3_, cm^−1^) υ_max_ = 1672, 1709, 1756, 3454; HRMS (ESI) *m/z*: [M+Na]^+^ Calcd for C_24_H_36_O_6_Na, 443.2396; found, 443.2404.

^1^H NMR (400 MHz, CDCl_3_, ppm): δ 6.95–6.99 (td, *J* = 6.84, 1.72 Hz, 1 H), 5.89–5.91 (d, *J* = 5.81, 1H), 4.84 (s, 1H), 4.52–4.56 (dd, *J* = 6.11, 11.25 Hz, 1H), 4.46 (s, 1H), 4.16–4.21 (dd, *J* = 1.71, 11.0 Hz, 1H), 4.13–4.17 (d, *J* = 11.0 Hz, 1H), 3.41–3.45 (m, 1H), 3.27–3.29 (d, 1H), 2.38–2.45 (m, 2H),2.30–2.34 (m, 3H), 1.94–1.98 (m, 1H), 1.78–1.82 (m, 4H), 1.62–1.70 (m, 4H), 1.17–1.28 (m, 7H), 0.92–0.96 (m, 3H), 0.63 (s, 3H).

^13^C NMR (100 MHz, CDCl_3_, ppm): δ 173.21, 169.13, 150.47, 146.65, 123.83, 108.80, 80.24, 71.71, 67.51, 64.04, 55.72, 55.08, 42.73, 38.72, 37.62, 36.93, 35.86, 28.03, 25.23, 23.60, 22.68, 18.33, 15.04, 13.59.

*Andrographolide-14-caproate (5)*.

Colourless semi-solid; m.p. 105.9–108.2 °C; IR (CHCl_3_, cm^−1^) υ_max_ = 1673, 1740, 1752, 3389; HRMS (ESI) *m/z*: [M+1] ^+^ Calcd for C_30_H_49_O_6_, 505.3520; found, 505.3524.

^1^H NMR (400 MHz, CDCl_3_, ppm): δ 6.91–6.95 (td, *J* = 6.84, 1.72 Hz, 1H), 5.85–5.88 (d, *J* = 5.81, 1H), 4.82 (s, 1H), 4.44–4.55 (m, 2H), 4.09–4.18 (m, 2H), 3.38–3.46 (m, 1H), 3.24–3.46 (m, 1H), 2.26–2.39 (m, 4H), 1.72–1.81 (m, 4H), 1.51–1.65 (m, 3H), 1.16–1.28 (m, 22H), 0.75–0.87 (m, 4H), 0.60 (s, 3H).

*^13^C NMR* (100 MHz, CDCl_3_, ppm): δ 173.44, 169.15, 150.48, 146.61, 123.86, 108.86, 80.33, 71.73, 67.52, 64.06, 55.73, 55.08, 42.80, 38.74, 37.63, 36.92, 34.08, 31.86, 29.37, 29.29, 29.17, 29.08, 28.19, 28.08, 25.25, 24.89, 23.61, 22.65, 15.08, 14. 10.

*Andrographolide-14-laurate (6)* values matched with reported data [2].

Colourless solid; m.p. 109.2–111.4 °C; **IR** (CHCl_3_, cm^−1^) υ_max_ = 1671, 1726, 1743, 3346; HRMS (ESI) *m/z*: [M+Na]^+^ Calcd for C_32_H_52_O_6_ Na, 555.3647; found, 555.3656.

^1^H NMR (400 MHz, CDCl_3_, ppm): δ 6.95–7.02 (td, *J* = 6.84, 1.72 Hz, 1H), 5.89–5.92 (d, *J* = 5.81, 1H), 4.86(s, 1H), 4.48–4.59 (m, 2H), 4.13–4.23 (m, 2H), 3.28–3.51 (m, 3H), 2.30–2.44 (m, 5H), 1.76–1.86 (m, 4H), 1.53–1.69 (m, 3H), 1.12–1.36 (m, 23H), 0.80–0.94 (m, 4H), 0.65 (s, 3H).

*^13^C NMR* (100 MHz, CDCl_3_, ppm): 173.47, 169.18, 150.51, 146.64, 123.89, 108.89, 80.36, 71.76, 67.55, 64.09, 55.76, 55.11, 42.83, 38.77, 37.66, 36.95, 34.10, 31.89, 29.96, 29.58, 29.40, 29.32, 29.19, 29.11, 28.22, 28.11, 25.27, 24.92, 23.63, 22.68, 15.11, 14.13.
