# Supplementary material for: Lipase catalyzed synthesis of antimicrobial andrographolide derivatives
Source: Data Brief. 2018 Mar 28;18:1134–41. doi: 10.1016/j.dib.2018.03.103 (PMC5996720; doi:10.1016/j.dib.2018.03.103)
Supplement: Supplementary file 1 — Supplementary material [file mmc2.pdf]

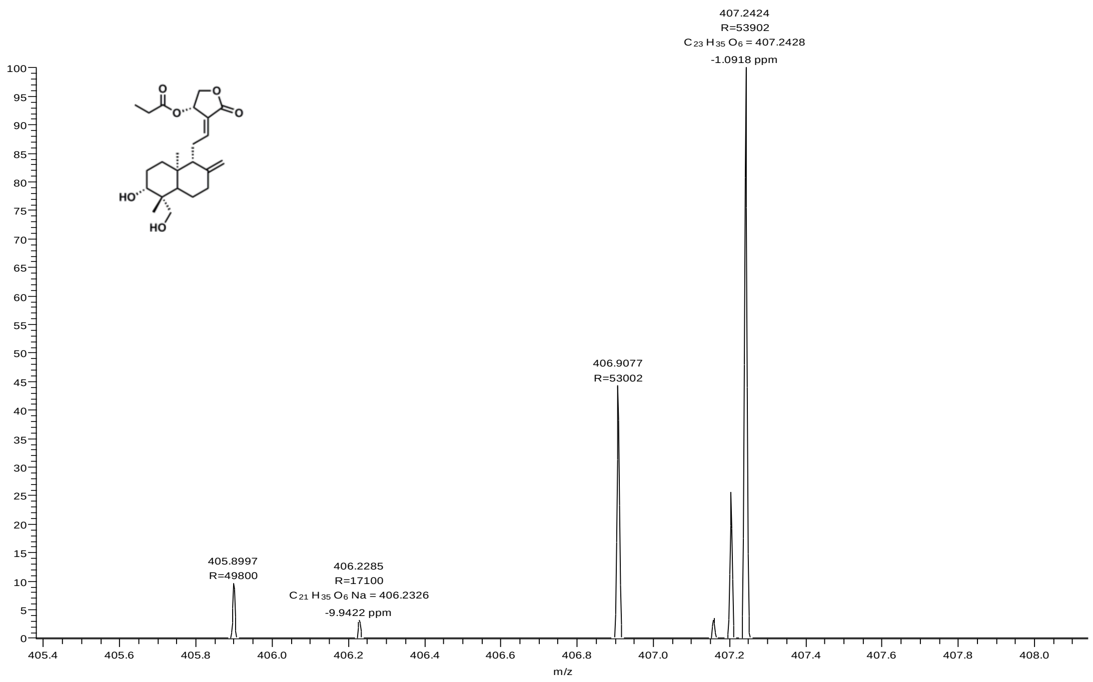

Figure S1a. High-Resolution Mass spectra of andrographolide-14-propionate (**3**)

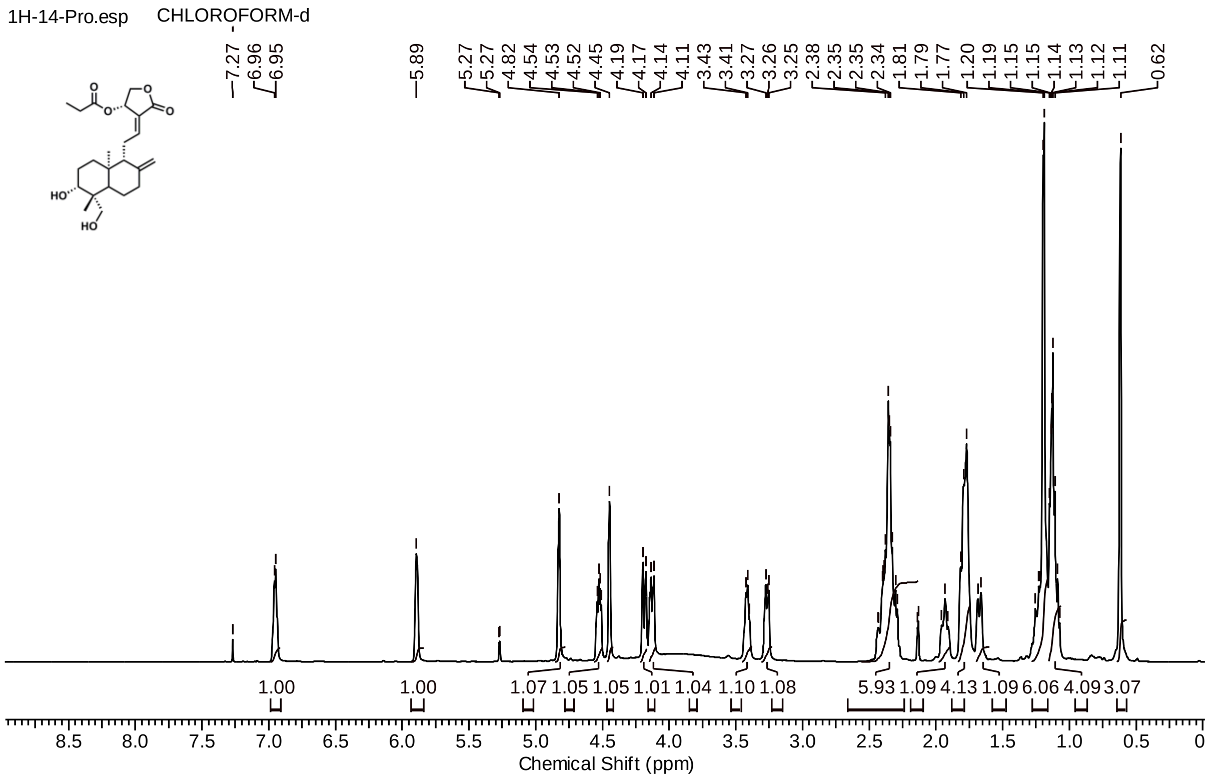

Figure S1b. ^{1}H- NMR spectrum of andrographolide-14-propionate (**3**).

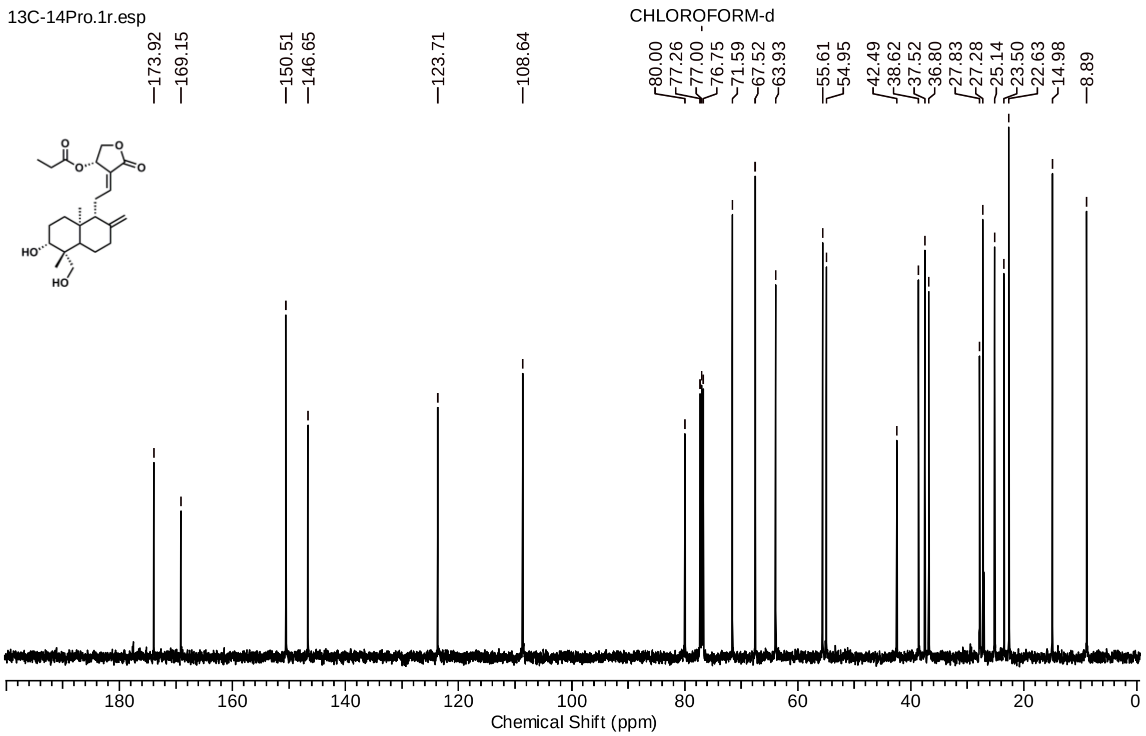

Figure S1c. ^{13}C- spectra of andrographolide-14-propionate (**3**).

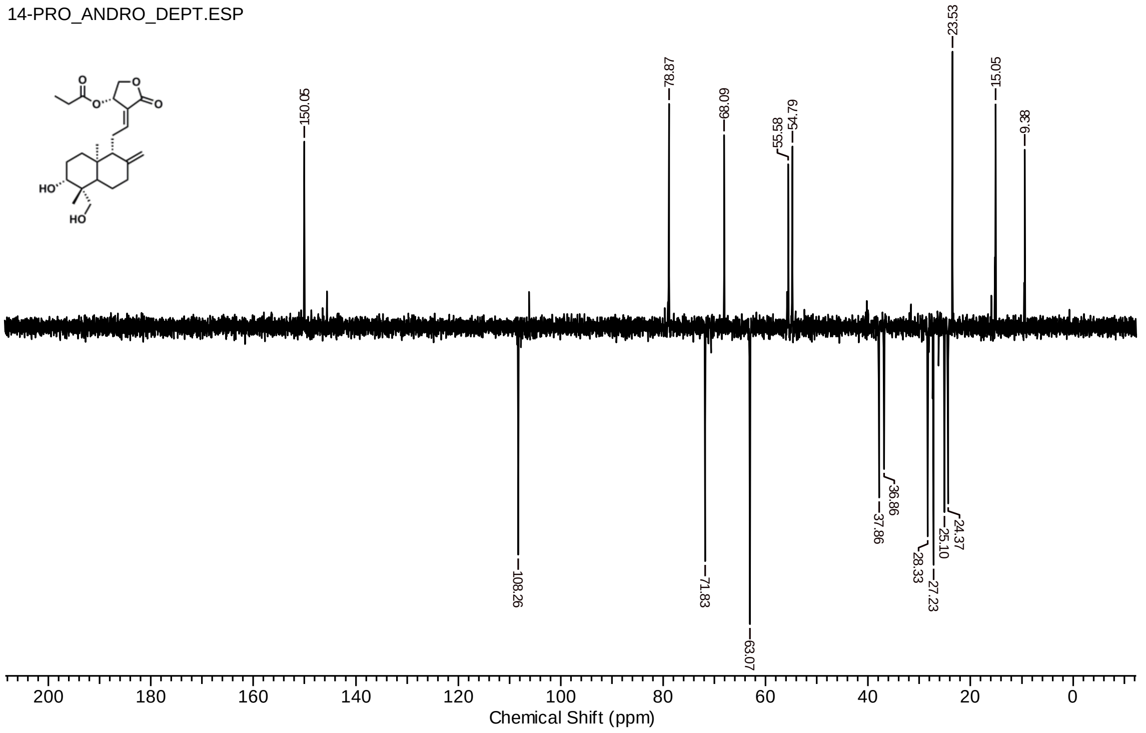

Figure S1d. Distortionless enhancement by polarization transfer (DEPT) NMR spectrum of andrographolide-14-propionate (**3**).

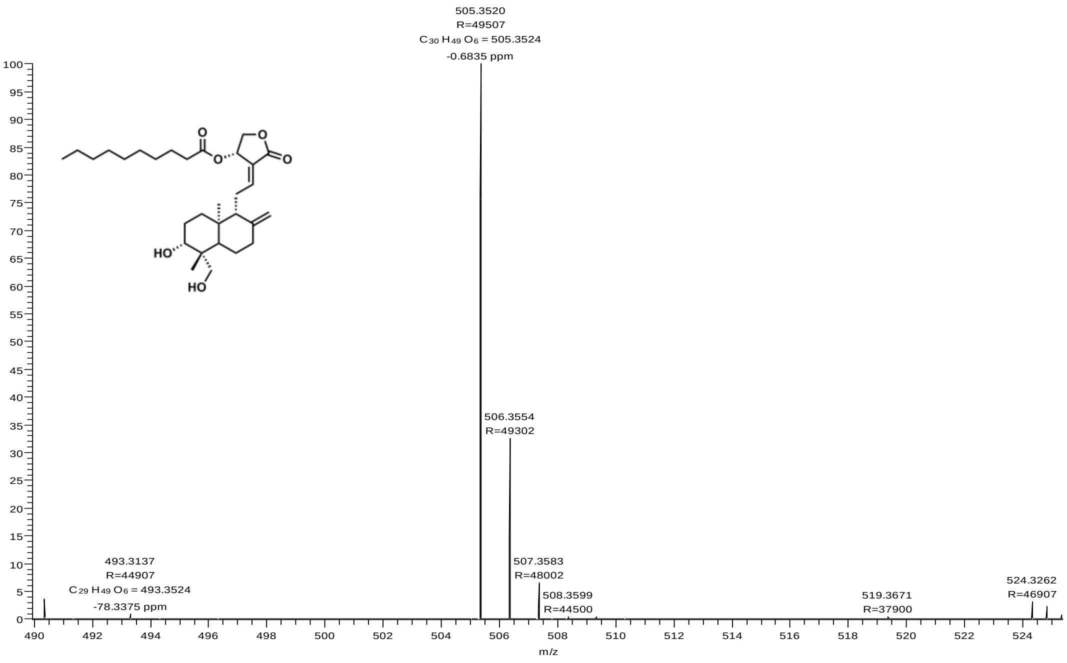

Figure S2a. High-Resolution Mass spectra of andrographolide-14- caproate (**5**)

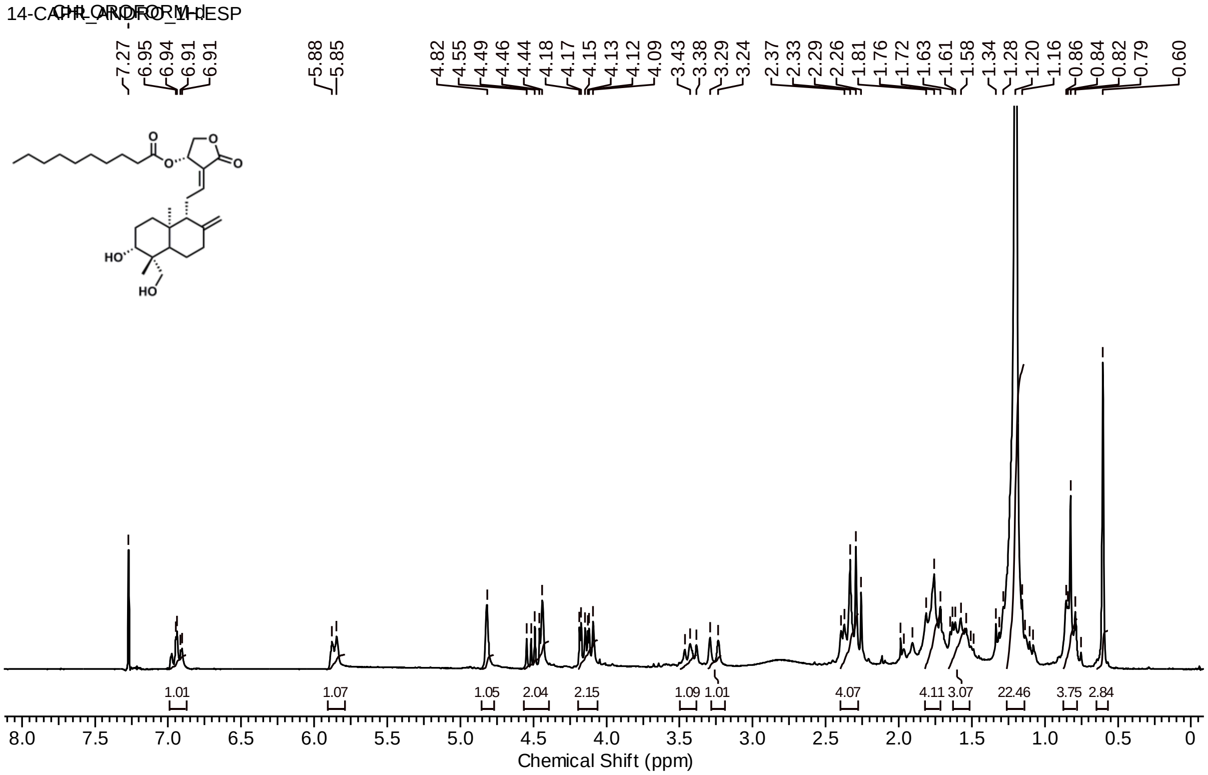

Figure S2ab. ^{1}H- NMR spectrum of andrographolide-14-caproate (**5**)

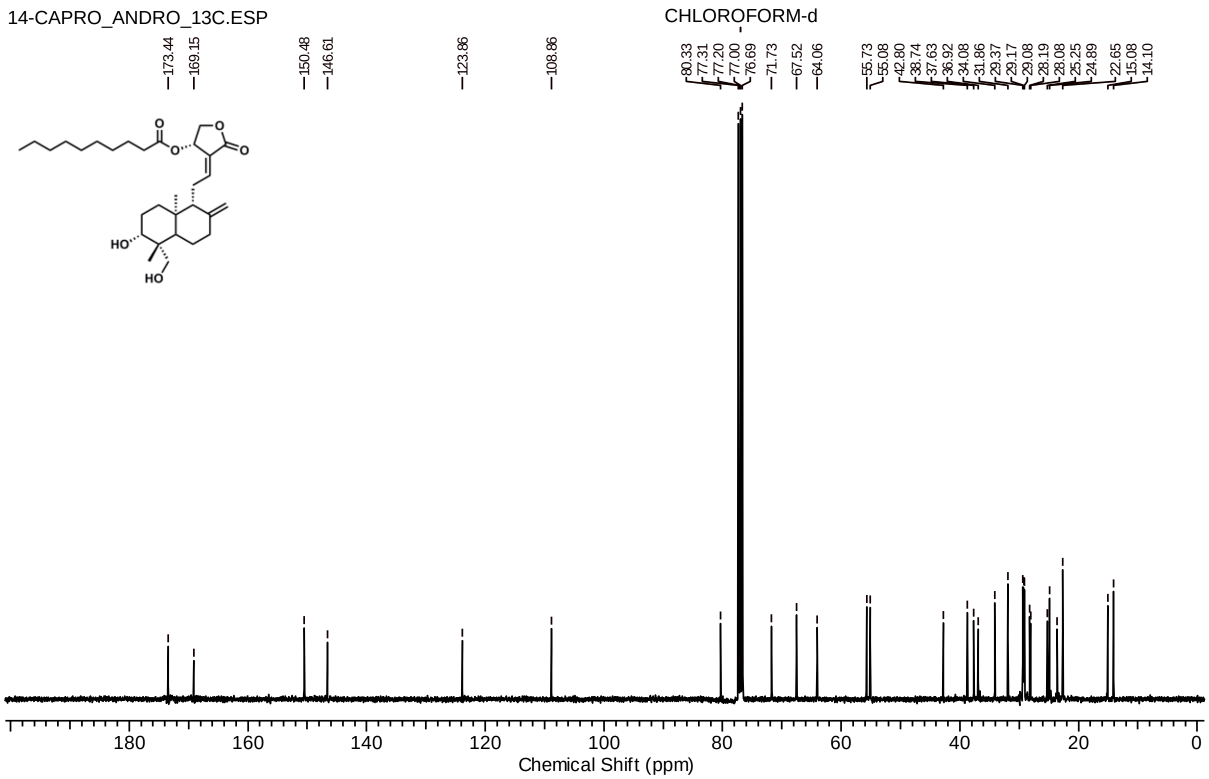

Figure S2c. ^{13}C- spectra of andrographolide-14-caproate (**5**)

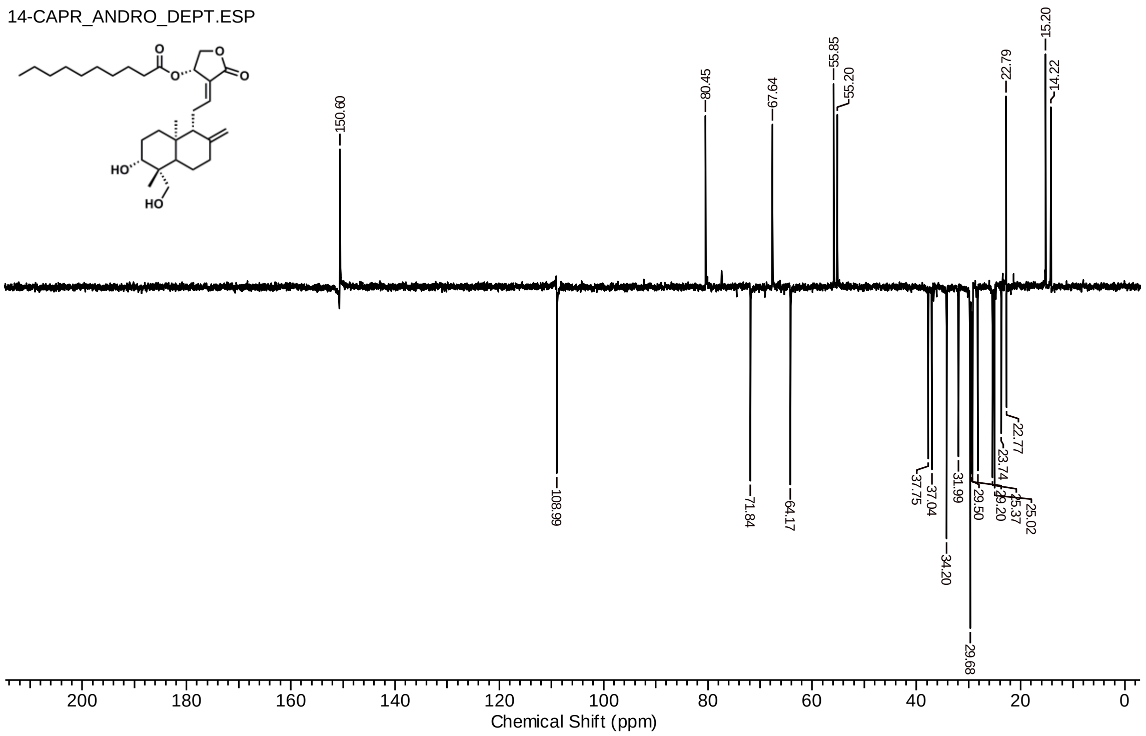

Figure S2d. Distortionless enhancement by polarization transfer (DEPT) NMR spectrum of andrographolide-14-caproate (**5**)
